# Improving malaria knowledge and practices in rural Myanmar through a village health worker intervention: a cross-sectional study

**DOI:** 10.1186/1475-2875-13-5

**Published:** 2014-01-04

**Authors:** Moh Moh Lwin, May Sudhinaraset, Aung Kyaw San, Tin Aung

**Affiliations:** 1 Population Services International/Myanmar, Yangon, Myanmar; 2 Global Health Group, University of California, San Francisco, San Francisco, CA, USA

**Keywords:** Social franchise programme, Rural, Community health workers, Malaria, Intervention

## Abstract

**Background:**

Since 2008 the Sun Primary Health (SPH) franchise programme has networked and branded community health workers in rural Myanmar to provide high quality malaria information and treatment. The purpose of this paper is to compare the malaria knowledge level and health practices of individuals in SPH intervention areas to individuals without SPH intervention

**Methods:**

This study uses data from a cross-sectional household survey of 1,040 individuals living in eight rural townships to compare the knowledge level of individuals in SPH intervention areas to individuals without SPH intervention.

**Results:**

This study found that the presence of a SPH provider in the community is associated with increased malaria knowledge and higher likelihood of going to trained providers for fevers. Furthermore, the study found a dose–response, where the longer the duration of the programme in a community, the greater the community knowledge level.

**Conclusion:**

The study suggests that community health workers might have significant impact on malaria-related mortality and morbidity in rural Myanmar.

## Background

Malaria causes approximately 500 million infections and about 650,000 deaths every year worldwide [1,2]. In Myanmar, an estimated 37 million people live in malaria endemic areas, where 70% of malaria-risk people live in rural areas [2]. Late diagnosis and inappropriate treatment of malaria can lead to complications and death [3,4]. Early diagnosis and effective treatment can be improved through the use of health workers. Numerous programmes in low- and middle-income countries that utilized trained health workers in rural settings demonstrated improvements in rural populations’ access to accurate diagnosis and treatment [5-11]. Since 2004, community-based malaria control projects have gained popularity in Myanmar. For example, village health volunteers in 160 remote villages provided insecticide-treated mosquito nets (ITNs) and early diagnosis and appropriate treatment, resulting in reported positive health impacts [6,12].

Health workers help to improve early diagnosis and effective treatment for malaria by increasing access to and provision of quality services and products such as RDTs (rapid diagnostic test kits) and ACT (artemisinin-based combination therapy). RDTs provide an easy and accurate confirmation of symptomatic diagnosis of malaria in resource-poor settings [9,10,13]. In several studies conducted in Thailand, Ethiopia, and Uganda, community-based malaria care services combined with RDT use have been shown to reduce malaria transmission and lower malaria morbidity and mortality in rural populations [10,14,15]. ACT is the most effective for uncomplicated malaria cases, using RDT to correctly diagnose patients reduces presumptive treatments, limiting the overuse of ACT, lowering programme costs, and delaying the emergence of drug resistance [10,13,16,17].

### Intervention

**Figure 1:**
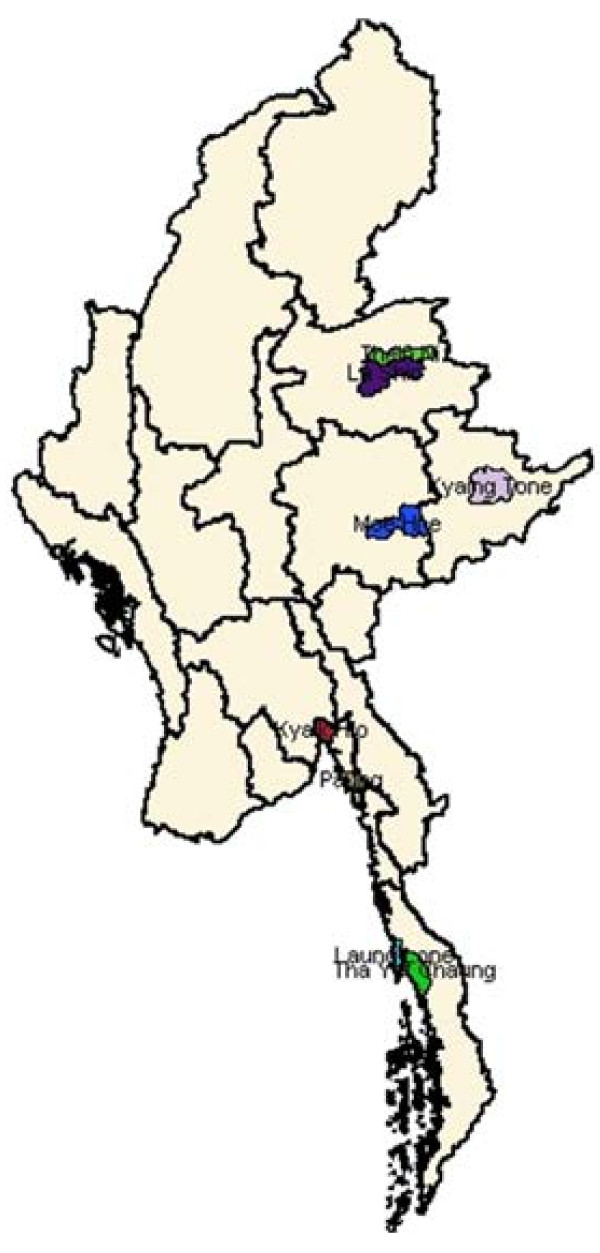
Map of SPH Interventions of 8 rural townships in Myanmar.

Launched in 2008 in eight townships, the Sun Primary Health (SPH) network was scaled up in 2009 to 19 townships where 338 SPH providers were trained, 20 townships in 2010 where 280 SPH were supported for malaria services, and 37 townships in 2011 where 557 SPH providers were requested to give malaria services to their communities (see Figure 1). The SPH malaria programme was implemented according to areas in need and getting appropriate approval from local division directors or township medical officer.

As part of SPH, PSI/Myanmar and the Myanmar government township medical officers recruited volunteer health workers to provide basic health services such as diagnosis and treatment of uncomplicated malaria cases. Health workers recruited into SPH were trained and provided with RDTs for malaria diagnosis and differentiation of *Plasmodium falciparum*, *Plasmodium vivax* and/or mixed infection. Training topics covered malaria counseling, malaria transmission, use of RDTs, treatment, and prevention and control of malaria by using impregnated bed nets. Health workers were also awarded with performance-based incentive schemes based on malaria tested or treated cases per month.

While the SPH network has served thousands of populations, the impact of the programme in increasing community knowledge and services is unknown. The study analysed data from a cross-sectional household survey of 1,040 individuals living in eight townships of rural areas to compare the knowledge level of individuals in SPH intervention areas to individuals without SPH intervention. The study hypothesized that individuals living in SPH communities have higher knowledge and correct treatment levels compared to non-SPH areas.

## Methods

### Recruitment and study subjects

The cross-sectional quantitative household survey was conducted by researchers from Population Services International/Myanmar (PSI/M) in January 2012 and recruited 2,080 study participants from eight townships in five states/divisions including Mon, Eastern Shan, Northern Shan, Southern Shan and Tanintaryi Division. Townships were randomly selected from a list of 92 townships classified as malaria endemic townships according to Unicef and the Vector Borne Disease Control Team (VBDC) in Myanmar. The survey covered 11,491 communities in 2009, 21,973 communities in September/October 2011, and 2,041 communities in December 2011, and the primary sampling unit was households. In total, the analysis included 1,040 respondents. The inclusion criteria was 18 years of age or older, and caregivers were asked about children’s fever in the last two weeks.

### Measurements

The primary outcome of interest was malaria knowledge and treatment. The study measured malaria knowledge with two indicators: knowledge on prevention of malaria by using ITNs and ever heard of malaria RDT (all binary outcomes, yes/no). The study measured malaria treatment categorically by whether they receive treatment from trained providers, buy and take medicine from drug store, or take treatment at home.

The main predictor of interest was whether individuals received the SPH intervention in their communities or did not receive SPH intervention, measured as a binary variable. The study calculated dose–response of the intervention by looking at the duration of the programme in communities (measured categorically from 2009, 2010, 2011). Demographic characteristics include age of study participations (categorical); education level (categorical); gender (binary); and occupation (categorical).

### Analyses

Four sets of analyses were conducted. First, simple chi-2 statistics and t-tests were used to test statistical differences of demographic characteristics between SPH versus non-SPH communities. Second, bivariate analyses were used to test associations between demographic characteristics and the malaria knowledge and treatment. Third, multivariable analyses were conducted to control for potential confounders. Due to lack of statistical significance of demographic characteristics across SPH and non-SPH communities (Table 1), only bivariate analyses are shown in the paper. Lastly, to test for dose–response effects of the intervention, the analysis stratified provider malaria knowledge and treatment outcomes by the timing of intervention, or duration in which the intervention has been in the community (i.e. 2009, Sep – Oct 2011, Dec 2011). The study stratified all analyses by SPH versus non-SPH communities and used STATA 12MP.

**Table 1:** Demographic characteristics of household members by SPH vs non-SPH communities

	**SPH communities (n = 321)**	**Non-SPH communities (n = 719)**	**Chi2, p-value**	**Total (N = 1040)**
**Age (years)**				
15 – 35	104 (32.40)	227 (31.57%)		331 (31.83%)
35 – 50	103 (32.09%)	245 (34.08%)		348 (33.46%)
50 – 65	88 (27.41%)	202 (28.09%)	1.4484, 0.694	290 (27.88%)
>65	26 (8.10%)	45 (6.26%)		71 (6.83%)
**Education Level**				
No schooling	85 (26.48%)	212 (29.49%)		297 (28.56%)
Middle school	224 (69.78%)	461 (64.12%)	4.6095,0.2030	685 (65.87%)
High School	10 (3.12%)	39 (5.42%)		49 (4.71%)
Graduate School	2 (0.62%)	7 (0.97%)		9 (0.87%)
**Gender**				
Female	235 (73.21%)	486 (67.59%)	3.2902, 0.070	721 (69.33%)
Male	86 (26.79%)	233 (32.41%)		319 (30.67%)
**Occupation**				
Higher management	3 (0.93%)	5 (0.70%)	5.9039, 0.116	8 (0.77%)
Own business/shopkeeper	20 (6.23%)	70 (9.74%)		90 (8.65%)
Manual labourer	287 (89.41%)	631 (87.76%)		918 (88.27%)
Retirement	11 (3.43%)	13 (1.81%)		24 (2.31%)

Time of implementation:

2009;

2010;

2011 32(9.97%), 190(59.19%) and 99(30.84%).

### Ethical approval

Population Services International’s (PSI) Ethical Review Board approved this study.

## Results

In total, 1,040 participants were included in the study, with 321 respondents in communities that received an SPH and 719 respondents living in communities that did not have the intervention. Analyses of basic demographic characteristics suggest that there were no statistically significant differences between SPH and non-SPH communities. Approximately 32% of the study sample was between 15–35 years, 33% between the ages of 35–50 years, 28% between 50–65 years, and 7% older than 65 years (see Table 1). There was no statistically significant difference in age between SPH and non-SPH communities (p = 0.694). The majority of respondents had at least a middle school education or higher (approximately 75%), while 28.6% reported having no schooling. The majority of respondents in both the SPH and non-SPH communities were female (73.2% vs. 67.6%, respectively, p = 0.07). Overwhelmingly, respondents reported working in manual labor (88.3%), followed by owning a business/shopkeeper (8.7%), retired (2.3%), and less than 1% reported being in higher management (see Table 1).

Across a number of malaria knowledge and treatment-related questions, SPH communities scored higher compared to non-SPH communities. Respondents living in SPH communities were more likely to correctly identify using ITN as prevention of malaria (33.3% vs. 25.7%, p > 0.05), and more likely to have ever heard of RDTs for malaria (68.5% vs. 61.2%, p = 0.023). Moreover, respondents in communities with SPH providers were also more likely to be treated from a trained provider compared to those without a SPH provider (79.1% vs. 69.5%, p > 0.05). Respondents in non-SPH communities, on the other hand, were more likely to buy and take medicine from drug stores or take treatment at home compared to respondents in SPH communities (23% vs. 19%, p < 0.01) (see Table 2).

**Table 2:** Comparison of malaria knowledge and treatment between SPH vs. non-SPH

	**SPH communities (n = 321)**	**Non-SPH communities (n = 719)**	**Chi2, p-value**	**Total (N = 1040)**
**Key determinants of knowledge**				
Knowledge on prevention of malaria by using ITN	107 (33.33%)	185 (25.73%)	6.3529, 0.012	292 (28.08%)
Ever heard of malaria diagnostic blood test (RDT)	220 (68.54%)	440 (61.20%)	5.1558, 0.023	660 (63.46%)
**Intent to treat**				
Take treatment from trained providers	254 (79.13%)	500 (69.54%)	16.6445, 0.000	754 (72.50%)
Buy and take medicine from drug store	61 (19%)	165 (22.95%)		226 (21.73%)
Take treatment at home	6 (1.87%)	54 (7.51%)		60 (5.77%)

**Figure 2:**
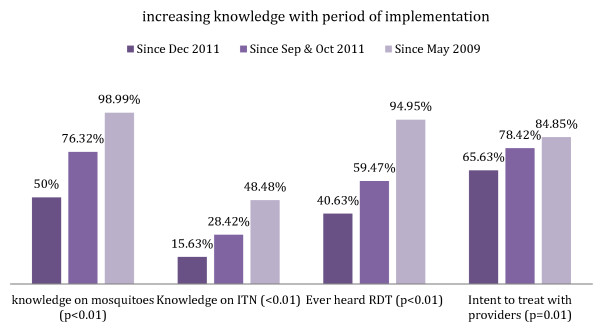
Duration of programme exposure and malaria knowledge.

The study found a dose–response effect -the longer the time the intervention had been in place in communities, the greater the malaria knowledge and treatment behaviours across a number of indicators (i.e. knowledge of mosquitoes as causes of malaria, ITN use, treatment with trained provider). For example, communities with a longer intervention period (2009) reported almost 49% knowledge of ITN use compared to only 15.6% of respondents with the shortest intervention period (2011). The two-year time period of the intervention increased the knowledge score by more than three times. This was the case across practically all knowledge and treatment indicators (see Figure 2).

## Discussion

This study was the first to evaluate the impact of the Sun Primary Health (SPH) intervention on malaria knowledge and behaviours among rural populations in Myanmar. The study found that the presence of a SPH provider in the community is associated with increased malaria knowledge, such as knowledge of use of ITN and RDT, and higher likelihood of going to trained providers for fevers. Lack of statistically significant differences in basic demographic characteristics between SPH and non-SPH groups strengthens the finding that there is an association between a SPH provider and higher knowledge levels. Furthermore, the study also found that communities with a longer duration of intervention (intervention introduced in 2009) had better knowledge score and treatment behaviours compared to communities with more recent introduction of providers (in 2011).

There are a number of limitations to the study. First, the study was not able to assess characteristics regarding patient-provider relationship, such as the quality of the providers, the frequency of visiting providers, or whether respondents interacted with SPH providers. Future studies should examine the quality of health workers and how that relates to patient practices and knowledge levels. Second, because the study uses cross-sectional data, the study cannot infer causality. However, these analyses take advantage of the stepped design of the intervention and examine dose–response, a criteria for causal inference.

Despite the limitations, this study demonstrates the critical and potential role of health workers in improving health in rural Myanmar. There is a dearth of studies on promising interventions in Myanmar, particularly in rural areas, and this study not only demonstrates a change in knowledge score between SPH and non-SPH communities, but also suggests that there are cumulative effects over time. Future studies should concentrate on a better understanding of the direct and indirect effects of SPH providers, how training impacts the quality of health workers, and explore whether greater frequency and contact with SPH providers result in better health outcomes. Moreover, future studies should concentrate on the health impact of communities, going beyond knowledge indicators to measure malaria prevalence, incidence, morbidity, and mortality. This study highlights the opportunity to engage with rural health workers in improving malaria knowledge, care, and practices.

## Consent

Written informed consent was obtained from the patient’s guardian/parent/next of kin for the publication of this report and any accompanying images.

## Competing interests

The authors declare that they have no competing interests.

## Authors’ contributions

ML carried out analyses and drafted the manuscript. MS oversaw data analysis and participated in drafting the manuscript. AS participated in the design of the study. TA conceived of the study, and participated in its design and coordination. All authors read and approved the final manuscript.

## References

[B1] Snow RW Guerra CA Noor AM Myint HY Hay SI The global distribution of clinical episodes of *Plasmodium falciparum* malaria Nature 2005 434 214 217 10.1038/nature03342 15759000 PMC3128492

[B2] WHO World malaria report 2012 2012 Geneva: World Health Organization

[B3] Byakika-Kibwika P Ndeezi G Kamya M Health care related factors associated with severe malaria in children in Kampala, Uganda Afr Health Sci 2009 9 206 210 20589153 PMC2887032

[B4] WHO Guidelines for the treatment of malaria 2010 Geneva: World Health Organization 25473692

[B5] Yasuoka J Poudel KC Poudel-Tandukar K Nguon C Ly P Socheat D Jimba M Assessing the quality of service of village malaria workers to strengthen community-based malaria control in Cambodia Malar J 2010 9 109 10.1186/1475-2875-9-109 20412600 PMC2873522

[B6] Ohnmar Tun-Min San-Shwe Than-Win Chongsuvivatwong V Effects of malaria volunteer training on coverage and timeliness of diagnosis: a cluster randomized controlled trial in Myanmar Malar J 2012 11 309 10.1186/1475-2875-11-309 22946985 PMC3488026

[B7] Lee CI Smith LS Shwe Oo EK Scharschmidt BC Whichard E Kler T Lee TJ Richards AK Internally displaced human resources for health: villager health worker partnerships to scale up a malaria control programme in active conflict areas of eastern Burma Glob Public Health 2009 4 229 241 10.1080/17441690802676360 19384681

[B8] Hawkes M Katsuva JP Masumbuko CK Use and limitations of malaria rapid diagnostic testing by community health workers in war-torn Democratic Republic of Congo Malar J 2009 8 308 10.1186/1475-2875-8-308 20028563 PMC2804690

[B9] Min-Naing C Gatton ML Performance appraisal of rapid on-site malaria diagnosis (ICT malaria Pf/Pv test) in relation to human resources at village level in Myanmar Acta Trop 2002 81 13 19 10.1016/S0001-706X(01)00189-9 11755428

[B10] Kyabayinze DJ Asiimwe C Nakanjako D Nabakooza J Counihan H Tibenderana JK Use of RDTs to improve malaria diagnosis and fever case management at primary health care facilities in Uganda Malar J 2010 9 200 10.1186/1475-2875-9-200 20624312 PMC2914063

[B11] Yeung S Damme WV Socheat D White NJ Mills A Access to artemisinin combination therapy for malaria in remote areas of Cambodia Malar J 2008 7 96 10.1186/1475-2875-7-96 18510724 PMC2430580

[B12] SEARO | MCC-WHO-3DF community-based malaria control: progress and challenges http://www.searo.who.int/myanmar/areas/mccwho3dfcommunitybasedmalaria/en/index.html

[B13] Skarbinski J Ouma PO Causer LM Kariuki SK Barnwell JW Alaii JA De Oliveira AM Zurovac D Larson BA Snow RW Rowe AK Laserson KF Akhwale WS Slutsker L Hamel MJ Effect of malaria rapid diagnostic tests on the management of uncomplicated malaria with artemether-lumefantrine in Kenya: a cluster randomized trial Am J Trop Med Hyg 2009 80 919 926 19478249

[B14] Carrara VI Sirilak S Thonglairuam J Rojanawatsirivet C Proux S Gilbos V Brockman A Ashley EA McGready R Krudsood S Leemingsawat S Looareesuwan S Singhasivanon P White N Nosten F Deployment of early diagnosis and mefloquine- artesunate treatment of falciparum malaria in Thailand: the Tak malaria initiative PLoS Med 2006 3 e183 10.1371/journal.pmed.0030183 16719547 PMC1470664

[B15] Lemma H Byass P Desta A Bosman A Costanzo G Toma L Fottrell E Marrast A-C Ambachew Y Getachew A Mulure N Morrone A Bianchi A Barnabas GA Deploying artemether-lumefantrine with rapid testing in Ethiopian communities: impact on malaria morbidity, mortality and healthcare resources Trop Med Int Health 2010 15 241 250 10.1111/j.1365-3156.2009.02447.x 19961564

[B16] Mutabingwa TK Artemisinin-based combination therapies (ACTs): best hope for malaria treatment but inaccessible to the needy! Acta Trop 2005 95 305 315 10.1016/j.actatropica.2005.06.009 16098946

[B17] WHO Marketing of oral artemisinin-based monotherapy medicineshttp://www.who.int/malaria/marketing_of_oral_artemisinin_monotherapies/en/

